# Design of Peptide-Based Nanovaccines Targeting Leading Antigens From Gynecological Cancers to Induce HLA-A2.1 Restricted CD8^+^ T Cell Responses

**DOI:** 10.3389/fimmu.2018.02968

**Published:** 2018-12-21

**Authors:** Sue D. Xiang, Kirsty L. Wilson, Anne Goubier, Arne Heyerick, Magdalena Plebanski

**Affiliations:** ^1^Department of Immunology, Faculty of Medicine, Nursing and Health Sciences, Central Clinical School, Monash University, Melbourne, VIC, Australia; ^2^PX Biosolutions Pty Ltd., South Melbourne, VIC, Australia; ^3^Ovarian Cancer Biomarker Laboratory, Hudson Institute of Medical Research, Clayton, VIC, Australia; ^4^School of Health and Biomedical Sciences, RMIT University, Bundoora, VIC, Australia

**Keywords:** nanoparticles, HPV, WT1, survivin, CD8 T cell epitopes, vaccine, immunogenicity, HLA-A2.1

## Abstract

Gynecological cancers are a leading cause of mortality in women. CD8^+^ T cell immunity largely correlates with enhanced survival, whereas inflammation is associated with poor prognosis. Previous studies have shown polystyrene nanoparticles (PSNPs) are biocompatible, do not induce inflammation and when used as vaccine carriers for model peptides induce CD8^+^ T cell responses. Herein we test the immunogenicity of 24 different peptides, from three leading vaccine target proteins in gynecological cancers: the E7 protein of human papilloma virus (HPV); Wilms Tumor antigen 1 (WT1) and survivin (SV), in PSNP conjugate vaccines. Of relevance to vaccine development was the finding that a minimal CD8^+^ T cell peptide epitope from HPV was not able to induce HLA-A2.1 specific CD8^+^ T cell responses in transgenic humanized mice using conventional adjuvants such as CpG, but was nevertheless able to generate strong immunity when delivered as part of a specific longer peptide conjugated to PSNPs vaccines. Conversely, in most cases, when the minimal CD8^+^ T cell epitopes were able to induce immune responses (with WT1 or SV super agonists) in CpG, they also induced responses when conjugated to PSNPs. In this case, extending the sequence around the CD8^+^ T cell epitope, using the natural protein context, or engineering linker sequences proposed to enhance antigen processing, had minimal effects in enhancing or changing the cross-reactivity pattern induced by the super agonists. Nanoparticle approaches, such as PSNPs, therefore may offer an alternative vaccination strategy when conventional adjuvants are unable to elicit the desired CD8^+^ T cell specificity. The findings herein also offer sequence specific insights into peptide vaccine design for nanoparticle-based vaccine carriers.

## Introduction

Gynecological malignancies, including ovarian, endometrial, vulvar, fallopian tube and cervical cancers, are the leading cause of mortality in women (~9.8% of cancer related deaths in women) (1), with the most lethal malignancy being ovarian cancer (2, 3). There are many factors that cause gynecologic cancers. Although oncogenes and tumor suppressor genes promote the growth of cancer, almost all cervical cancers and some cancers of the vagina and vulva are caused by a virus known as Human Papillomavirus (HPV). The development of a preventive vaccine to limit the infectivity and transmission of the HPV, working primarily through the induction of virus neutralizing antibodies, is a tremendous positive step forwards, but is not able to be used therapeutically (4–6). Moreover, there are also no licensed vaccines to target and treat the other gynecological malignancies, such as to ovarian cancer.

High levels of tumor infiltrating CD8^+^ T cells are associated with increased survival in patients with diverse gynecological malignancies, notably, with ovarian cancer (7, 8). Emerging immunotherapies which can re-establish full functionality for CD8^+^ T cells in the local tumor microenvironment, based primarily on disrupting immunosuppressive PD1/PDL1 interactions, are showing great promise in multiple clinical trials, and have been touted as a game-changer for cancer treatment (9). These advances are bringing renewed interest in the development of practical methods to increase initial CD8^+^ T cell numbers to relevant tumor antigens by vaccination. An additional major emerging trend for cancer immunotherapy is the ability to use high-throughput analysis “omics” techniques, such as transcriptomics, to define tumor subtypes and cancer cell heterogeneity (10, 11). These findings are being used to identify subtypes and hence patients most able to respond clinically to specific chemotherapies, an aspect of “precision” or “personalized” medicine. These omics techniques are also resulting in databases rich in antigen sequences, and are potentially able to define the best target antigens expressed by cancer cells within each patient, and to develop personalized vaccines.

Peptides offer a practical source of antigen for personalizing therapeutic cancer vaccines to induce high levels of CD8^+^ T cells. They are also non-infectious, completely defined, relatively easy to produce, and are generally considered to be safe. The design of peptide-based vaccines, particularly those involving new generation nanoparticle-based delivery systems, involves the challenge of ensuring correct antigen processing into MHC class I (MHC I) restricted epitopes to promote CD8^+^ T cell priming. Controversy remains in the literature on the nature of the peptides to be used in such vaccines in the context of cancer, ranging from (1) peptides representing only minimal native CD8^+^ T cell epitopes; (2) their agonist variants (to help break potential tolerance, or enhance MHC I binding or immunogenicity of peptides representing weak natural epitopes); (3) minimal peptide epitopes with added amino acids at either end, to promote stability in micro-environments which contain exopeptidases, as well as potentially promote appropriate cleavage or processing if the minimal epitopes are covalently conjugated to a nanoparticle; 4) the inclusion of CD4^+^ T cell epitopes, either by replicating in a peptide region from a protein that contains both CD4^+^ and CD8^+^ T cell epitopes, or constructing artificial constructs encompassing in one peptide containing CD8^+^ and CD4^+^ epitopes from different proteins. Further in this context, another limitation of peptide-based vaccines/immunotherapy is the need for each immune dominant epitope to match the patient's human leukocyte antigen (HLA). HLA polymorphisms in patients make it difficult to develop a peptide-based vaccine that are broadly applicable across the patient population.

The usually low immunogenicity of cancer associated antigens (which are often overexpressed or variant self-antigens) also needs the selection of powerful vaccine adjuvants and carriers able to promote strong immune responses. We have previous reported that nanoparticles at a specific size (~50 nm) induce strong immune responses when covalently linked to an antigen (12–14). As a platform technology, the specific size defined polystyrene nanoparticles (PSNPs) have shown powerful self-adjuvanting properties when used to deliver protein model antigens such as ovalbumin (OVA) (12), DNA plasmids expressing OVA (15), as well as high affinity peptides (13, 16), including strong antigens from respiratory syncytial virus (RSV) (17) and malaria liver stage antigens (16, 18). In these studies, PSNPs showed superior adjuvancity to conventional pro-inflammatory adjuvants such as Aluminum hydroxide (Alum), Quil A and monophosphoryl lipid A (MPL) for the induction of antigen specific CD8^+^ T cell and CD4^+^ T cells, particularly IFN-γ producing T cells, as well as long lasting antibody levels. A unique feature of the PSNP adjuvanting system is that, in contrast to other adjuvants which work by promoting inflammation via toll-like-receptors (TLRs) or pathogen-recognition-receptors (PRRs) signaling, PSNPs do not induce conventional inflammation (mediated by Erk or Akt signaling) (19), or the induction of conventional pro-inflammatory cytokines such as IL-6 and TNF (20), or the expansion of inflammation reactive regulatory T cells (Tregs) (18). These features could make these, and other systems with similar properties, particularly useful for the development of cancer therapeutic vaccines, where both inflammation and Treg induction are associated with tumor progression (21, 22).

Furthermore, our PSNPs-peptide vaccine formulations have also shown protective and therapeutic efficacies in various murine tumor models with multiple diverse peptide antigens [(12, 13, 15) and unpublished]. However, a major challenge in translation remains in understanding the rules by which to select useful peptides that can be appropriately processed and presented to stimulate CD8 T cell immunity. In this paper we specifically explore this challenge by testing >20 different peptide formulations in HLA-A2.1 transgenic animals. We hypothesized here that PSNPs could be effectively linked (covalently conjugated) to peptide antigens derived from gynecological tumors and generate immunogenic constructs capable of inducing HLA-A2.1 restricted CD8^+^ T cells. Moreover, herein we explore the diverse formulation challenges using peptides in vaccines generally, and specifically differences in processing into minimal CD8^+^ T cell epitope using nanoparticle-based vaccine such as PSNPs. To explore this issue, we studied diverse peptides derived from three different antigens associated with major and diverse gynecological malignancies: the E7 protein from HPV16, a demonstrated major target for CD8^+^ T cells in cervical cancer (23–25); Survivin (SV), an oncogenic inhibitor-of-apoptosis protein expressed in cervical and ovarian malignancies (26–32); and Wills Tumor antigen 1 (WT1), a well-studied antigen in the context of diverse tumor types such as leukemia and ovarian cancer (33) [reviewed by (34–36)]. WT1 has recently been listed among the top of the 75 ideal cancer antigens in immunotherapies by the U.S. National Cancer Institute (37).

## Materials and Methods

### Peptides and Carrier/Adjuvants

Table 1 lists all the peptides synthesized for this study. Peptide HPV01, HPV05, HPV08, SV01, SV02, and WT1B were synthesized by Auspep (Tullamarine, VIC, Australia); peptides HPV12, SV03 to SV09, WT1A, WT1C, WT1D, and WT1E were synthesized by CS Bio (Menlo Park, CA, United States). The purity (>95%) and identity of peptides were determined by HPLC and mass spectrometry, respectively.

**Table 1:** Peptides and sequences.

**Peptide code**	**Sequence**	**Amino acid position**
**HPV PEPTIDE ANTIGENS**		
HPV01	LLMGTLGIVCPICKQQLLRREVYDFAFRDLCIVYRDGN	HPV16-E7_82−94_ and HPV16-E6_41−65_
HPV05	TLGIVCPI	HPV16-E7_86−93_
HPV08	VQSTHVDIRTLEDLLMGTLGIVCPI	HPV16 E7_69−93_
HPV12	KQQLLRREVYDFAFRDLCIVYRDGN	HPV16-E6_41−65_
**WT1 PEPTIDE ANTIGENS**		
WT1A	RMFPNAPYL	WT1_126−134_
WT1B	YMFPNAPYL	WT1_126−134_ variant
WT1C	SGQAYMFPNAPYLPSCLES	WT1_122−140_
WT1D	AAYYMFPNAPYL	AAY+ WT1_127−134_
WT1E	AAYYMFPNAPYLPSCLES	AAY+WT1_127−134_ +PSCLES
**SURVIVIN (SV) PEPTIDE ANTIGENS**		
SV01	KKQFEELTLGEFLKLDRERAKNKIAKETNNKKKEF	SV_90−124_
SV02	GAPTLPPAWQPFLKDHRISTFKNWPFLEGCACTPE	SV_2−36_
SV03	ELTLGEFLKL	SV_95−104_
SV04	LTLGEFLKL	SV_96−104_
SV05	TLPPAWQPFL	SV_5−14_
SV06	RISTFKNWPFL	SV_18−28_
SV07	LTLGEFLKLDRERAKN	SV_96−111_
SV08	WQPFLKDHRISTFKN	SV_10−24_
SV09	HRISTFKNWPFLEGCACT	SV_17−34_
SV10	LMLGEFLKL	SV_96−104_ variant
SV11	ELMLGEFLKL	SV_95−104_ variant
SV12	DLAQMFFCFKELEGW	SV_53−67_ variant
SV13	KKQFEELMLGEFLKL	SV_90−104_ variant
SV14	KKQFEELMLGEFLKLDRERAK	SV_90−110_ variant
SV16	AAYLMLGEFLKL	AAY+SV10 (SV_96−104_ variant)

### Conjugating Peptide Antigen Onto Nanoparticles (PSNPs)

Selected antigen peptides (from Table 1) were chosen as peptide-based vaccine targets to form nanovaccine formulations. Each of the individual peptides were covalently conjugated to 40–50 nm carboxylated polystyrene nanoparticles (PSNPs, Polysciences Inc., Warrington, PA, United States) to form peptide-PSNPs vaccine formulations (e.g., HPV08-PSNPs, WT1B-PSNPs, or SV10-PSNPs etc.). Peptide conjugations were optimized for each peptide in order to achieve the best conjugation efficiency and size. In brief, following the conjugation procedures described previously (20), PSNPs at a final of 1% solids were pre-activated by gently mixing on a rotation wheel for 1 h at room temperature in a mixture containing 2-*N*-Morpholino-ethanesulfonic acid (MES) (50 mM final, pH = 6), 1-ethyl-3-(3-dimethylaminopropryl) carbodiimide hydrochloride (EDC) (4 mg/mL final) (Sigma-Aldrich, St. Louis, United States), *N*-hydrosulfosuccinimide (Sulfo-NHS) (50 mM final) (Pierce™, Thermo Fisher Scientific, Waltham, MA, United States) with final pH adjusted to be 5.5–6. After pre-activation, the excess activation agents (EDC and Sulfo-NHS) were removed from the pre-activation mix using a gel filtration column (Zeba spin desalting column following manufacturer's instruction, Thermo Fisher Scientific), and buffer exchanged at the same time via the column (buffer concentration and pH were optimized for each peptide antigen) before adding the peptide antigen for a further 2 h. The final conjugation mix was then dialysed against phosphate buffer (PBS, ~pH 7.2–7.4) in 1 kDa dialysis membrane (if non-PBS buffer was used as conjugation buffer). Final conjugation efficiency was determined by BCA™ protein assay (Pierce™ Micro BCA protein assay, Thermo Fisher Scientific) or amino acid analysis via HPLC (performed by Auspep). Particles sizing and polydispersity of the final peptide conjugated PSNPs (peptide-PSNPs) formulation were measured by dynamic light scattering (Zetasizer, Malvern Instruments Ltd, Worcestershire, United Kingdom). Each vaccine dose (100 μL) contained ~50 μg peptides and ~0.8–1% solid of PSNPs in PBS. The amounts of peptide antigen injected were matched for all formulations by adjusting the injection volume for each experiment. Those formulations were directly compared to the bench mark adjuvant CpG by direct mixing the testing peptides with CpG (20 μg/injection) (ODN 1826, InvivoGen, San Diego, CA, United States).

### Mice and Immunizations

The vaccine study was carried out in accordance with the recommendations of the “Institutional Guidelines and the Animal Welfare Assurance Act, Alfred Medical Research and Education Precinct (AMREP).” The protocol was approved by the AMREP animal ethics committee, Melbourne Australia. Immunogenicity of peptide-PSNPs vaccine formulations were tested in HLA-A2/Kb [A2KbC57BL/6JTgN(A2KbH2b)6Hsd)] transgenic mice (Animal Resources Centre, Western Australia). Briefly, mice (3–5/group) were immunized with testing formulations (~50–200 μl/injection) multiple times (as per experimental design) intradermally (i.d.) at the base of tail, 1–2 weeks apart (as per experimental design). Details of each immunization schedules are listed in the respective figure legends. Ten to Fourteen days following the last immunization, mice were euthanized by CO_2_ asphyxiation and spleens were removed and splenocytes were harvested and tested for antigen specific immunogenicity on an enzyme-linked immunospot (ELISpot) assay.

### ELISpot Assay

Antigen specific CD8^+^ T cell responses were evaluated by IFN-γ ELISpot assays (38). Briefly, 96-well filtration plates (MAHA, MSIP or MAIP plates, Millipore, Billerica, MA) were coated with 100 μl/well of anti-mouse IFN-γ (AN18, 5 μg/ml, MABTech, Stockholm, Sweden). Following overnight incubation at 4°C, the wells were washed and blocked with RPMI 1640 completed medium (CM) supplemented with 10% heat inactivated fetal bovine serum (FBS), 2 mM glutamine, 100 μg/ml streptomycin, 100 units/ml penicillin, 0.1 mM β-mercaptoethanol and 20 mM Hepes (all from Gibco, Life Technologies, CA, United States). Splenocytes (50 μl) from immunized mice (2 × 10^7^ cells/ml, either individual or pooled) were added to triplicate wells and incubated with 50 μl of recall antigens (see figure legends for specific details for respective experiment) at various concentrations (2.5–25 μg/ml final for all potential CD8^+^ epitopes and 25–100 μg/ml final for long peptides and protein) at 37°C incubator filled with 5% CO_2_ for a minimum of 16 h. Concanavalin A (Con-A) (1 μg/ml final, Amersham Biosciences, Uppsala, Sweden) was used as a positive control and background wells were added with CM only. The plates were then washed 6 times in PBS and incubated with 100 μl biotinylated detection antibodies [anti-mouse IFN-γ biotinylated mAb R4-6A2 (Mabtech) at 1 μg/ml final] at room temperature for 2 h. After washing as above, streptavidin-alkaline phosphatase was added (final at 1 μg/ml) and incubated for another 1.5 h at room temperature. Plates were then washed again, with a final wash using Reverse Osmosis (RO) water to remove residual PBS. The spots were developed using a colorimetric AP kit (Bio-Rad, Philadelphia, USA) following the manufacturers' instructions. Spot counting was performed using an AID ELISPOT Reader System (Autoimmun Diagnostika GmbH, Germany). The magnitudes of the IFN-γ induction in response to the recall antigen were compared either directly for its spot forming unit (SFU) or normalized against the background response (media alone response) from the same treatment group, calculated as stimulation index (SI) of SFU over background (SI = [SFU from the recall antigen stimulation in mice under the same treatment] / [SFU from the media alone stimulation in mice under the same treatment] for each corresponding recall antigens).

### Statistical Analysis

All statistical analyses were performed using Graph Pad Prism v6.04 software (Graph Pad Software, Inc., La Jolla, CA, United States) and Microsoft Excel (Microsoft Corporation, Redmond, WA, United States). Comparisons were performed using one or two-way ANOVA analysis as appropriate. Differences were considered statistically significant when *p* < 0.05. Values are expressed as mean ± standard deviation (SD).

## Results

The primary selection parameter for antigens capable of inducing CD8^+^ T cells in peptide-based cancer vaccine formulations is the ability of the peptide binding to MHC I molecules, and hence potential to be presented by appropriate antigen presenting cells (APC) to prime a CD8^+^ T cell response. The HLA-A2.1 molecule is the most common MHC-I molecule in humans (in ~44–50% of Caucasians and Asian) (39), and hence most initial vaccine development aims to identify suitable HLA-A2.1 restricted CD8^+^ T cell epitopes. CD4^+^ T cells may help to promote sustained CD8^+^ T cell reactivity, therefore when extending the peptide sequences around the desired CD8^+^ T cell minimal epitope, we took the opportunity to incorporate them together with CD4^+^ T cell epitopes with predicted broad binding affinity to HLA-DR, to offer a potential downstream powerful combination vaccine (40). However, the present study has only focused on the key issue of the generation of CD8^+^ T cell epitopes capable of inducing HLA-A2.1 restricted CD8^+^ T cell immunity in transgenic mice, since if this is not confirmed the vaccine combination would not go forwards into development for use in humans. Apart from epitope design, we also have considered that the peptides selected would need to be feasibly manufactured, as well as retain solubility and stability during the conjugation process (using EDC chemistry) to the vaccine carrier nanoparticles (PSNPs). To further help promote synthetic peptides being effectively processed into CD8^+^ or CD4^+^ T cell epitopes after attachment to the nanoparticles, as well as to help protect the peptide ends from the action of exoproteases present and also to improve the epitope recognition *in vivo*, in some cases, an extra region of amino acids was added at either or both ends (amino and carboxy) in the designed peptides.

Based on the above matrix of selection criteria, multiple peptides from HPV, Survivin and WT1 were designed, conjugated to nanoparticles and evaluated for their ability to induce antigen specific T cell responses, in particular CD8^+^ T cell responses. Further details that led to the design of specific peptides being synthesized, derived from each one of the three proteins, are expanded upon in each corresponding protein section below in results.

### HPV Peptide-Based Nanovaccine Formulations and Immunogenicity

#### HPV Peptide Antigen Design and Selection

HPV type 16 (HPV16) is responsible for up to 50% of all cervical cancers (41). HPV16 E7 is a protein of 98 amino acid (aa); highly immunogenic with good indications of clinical relevance and immunogenicity in cervical cancer (23–25). Based on extensive literature search (42–47), clinical trials (24, 25, 48) and manufacturing feasibility, as well as with the aids of epitope prediction programs (the predictive algorithm of the SYFPEITHI database: http://www.syfpeithi.de.), we designed and finalized three HPV peptide candidates as nanovaccine targets (Table 2): 1) HPV05: a HLA-A2.1-restricted minimal CD8^+^ T cell epitope (HPV16-E7_86−93_); 2) HPV01: a chimeric peptide consisting of two HLA-A2.1-restricted CD8^+^ T cell epitopes from HPV16-E7 (E7_82−94_) and a CD4^+^ T cell helper construct from HPV16-E6 (E6_41−65_) (HPV12); 3) HPV08: peptide fragment HPV16 E7_69−93_, containing both a CD4^+^ helper epitope and two HLA-A2.1-restricted CD8^+^ T cell epitopes. We also designed a peptide containing promiscuous CD4^+^ T cell epitopes (HPV12) as a helper peptide to be incorporated in some of the nanovaccine formulations when necessary.

**Table 2:** HPV peptide antigens (the predicted CD8^+^ T cell epitopes are underlined).

**Peptide code**	**Sequence**	**Amino acid position**	**Function**
HPV01	LLMGTLGIVCPICKQQLLRREVYDFAFRDLCIVYRDGN	HPV16-E7_82−94_, HPV16-E6_41−65_	Chimeric peptide consisting two HLA-A2.1-restricted CD8^+^ T cell epitopes from HPV16-E7_82−90, 86−93_ (47), and promiscuous HLA-DR restricted CD4^+^ T cell epitopes E_42−56_, _52−62, 54−68_ (49, 50)
HPV05	TLGIVCPI	HPV16-E7_86−93_	HLA-A2.1-restricted CD8^+^ T cell epitope.
HPV08	VQSTHVDIRTLEDLLMGTLGIVCPI	HPV16-E7_69−93_	Consists two HLA-A2.1-restricted CD8^+^ T cell epitopes HPV16-E7_82−90, 86−93_ (47) and a HLA-DRB1 CD4^+^ T cell epitope (HPV16-E7_73−87_) (50)
HPV12	KQQLLRREVYDFAFRDLCIVYRDGN	HPV16-E6_41−65_	Promiscuous HLA-DRB1 and HLA-DP0201 restricted CD4^+^ T cell epitopes (50)

#### Covalently Linking the HPV Peptide Candidates to Nanoparticles (PSNPs) and Optimization of Peptide-PSNPs Formulations

We have developed a procedure to covalently link the peptide antigens to nanoparticles and produce uniformly sized with single layer antigen attached nanovaccine formulations (20). The conjugation process requires the use of activating agents such as 1-ethyl-3-(3-dimethylaminopropryl) carbodiimide hydrochloride (EDC) and N-hydroxysulfosuccinimide (Sulfo-NHS) which cleaves the carboxyl groups and creates intermediate amine reactive ester bonds that allow covalent coupling of the peptide/proteins to the nanoparticles. This is best achieved in a condition of pH 5–6; however, at such pH, some peptides can be insoluble and form peptides/PSNPs aggregates, subsequently not suitable as nanovaccine formulations as particle size is crucial in particle-adjuvancity (38). Therefore, based on the standard procedure (see Material and Methods section), we altered conjugation conditions in the “conjugation step” and tested for a range of pH (5.5, 6, 6.5, 7 and 7.5) and buffers (PBS and NaHCO_3_) for each peptide candidate to ensure high conjugation efficiency as well as to minimize aggregations, since each peptide has its own physiochemical characteristics. The quality of the peptide conjugated nanoparticle formulations (peptide-PSNPs) were determined by sizes and polydispersity index (Pdl), as well as conjugation efficiency and antigen loading per particle.

**Figure 1:**
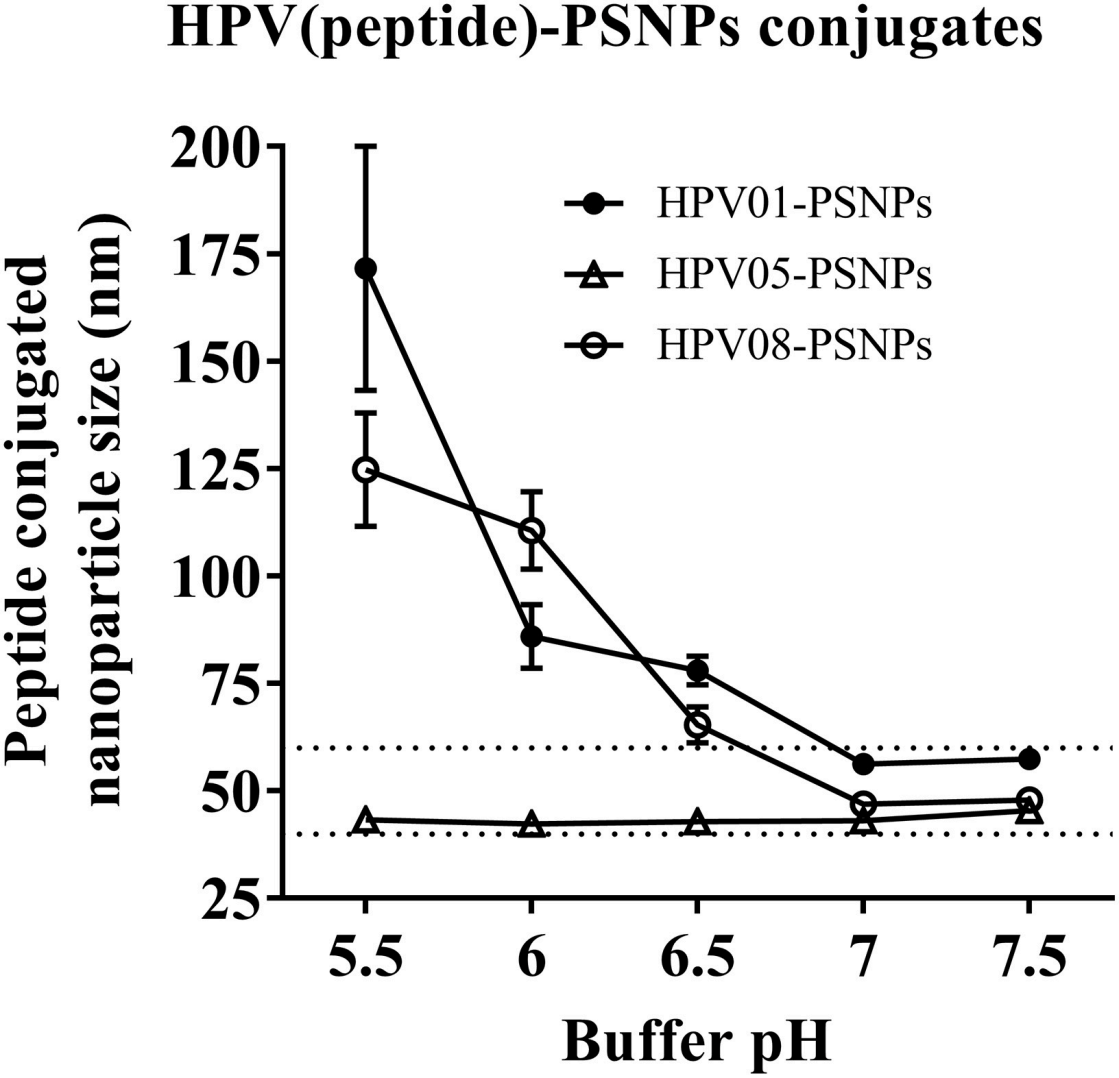
Optimization of conjugation conditions to covalently conjugate HPV peptides to PSNPs to produce uniform HPV(peptide)-PSNPs nanovaccine formulations. PSNPs (1% solid final) were pre-activated following the standard procedure (detailed in Materials and Methods), and then re-conditioned in different buffer and pH solutions before mixing with each peptide antigen (0.5 mg/ml final) for conjugation. After conjugation, the final particle sizes for each peptide-PSNPs formulation was assessed using a Zetasizer. Data presented as peptide-PSNPs conjugate size (nm) ± SD (3 repeated measurements) under each conjugation conditions for each peptide. The dotted lines indicated the acceptable nanovaccine formulation size range at 40–60 nm.

Conjugations of HPV peptides to the PSNPs were tested in PBS (for HPV01 and HPV08) and NaHCO_3_ (for HPV05) at the various pH. As results shown in Figure 1, at a lower pH 5.5–6.5 during the conjugation step, HPV(peptide)-PSNPs formulations tended to aggregate and increased in size, though the aggregations were reduced with the increasing pH, optimal at pH 7–7.5. The final pH range to generate acceptable sizes for all HPV(peptide)-PSNPs conjugates were selected on the basis of conditions which produce particle-conjugates in the range of 40–60 nm with nanoparticle polydispersity (Pdl) <0.2 (Table 3).

**Table 3:** Optimal conjugation conditions for the HPV(peptide)-PSNPs formulations.

**Peptide-PSNPs**	**Buffer**	**pH**	**Size (nm)**	**Polydispersity (Pdl)**	**Conjugation efficiency (%)**	**Antigen loading (peptide molecules/particle)**
HPV01-PSNPs	PBS	7.1	56.28 ± 0.68	0.23 ± 0.02	80^*^	4.36 × 10^2^
HPV05-PSNPs	50 mM NaHCO_3_	7.5	42.97 ± 0.34	0.08 ± 0.02	78^*^	2.72 × 10^3^
HPV08-PSNPs	PBS	7.5	48.34 ± 0.81	0.14 ± 0.01	100^*^	9.34 × 10^2^

* *Conjugation efficiency determined by HPLC amino acid analysis*.

To determine the conjugation efficiency under the selected optimal buffer and pH conjugation condition for each peptide tested here, the remaining non-binding peptide material in each formulation after the conjugation process was determined by BCA™ protein assay or analysis via HPLC where possible. The final conjugation efficiency was determined as the percentage of antigen successfully conjugated to PSNPs (the targeted antigen concentration was 0.5 mg/ml for all antigen peptides). Table 3 below summarizes the optimal conjugation conditions for each of the HPV peptide candidates evaluated in the study. The HPV05 peptide, representing the native HLA-A2.1-restricted minimal CD8^+^ T cell epitope (HPV16-E7_86−93_), achieved the highest antigen loading per PSNP (2.72 × 10^3^ peptide molecules/particle) compared to the other peptides, 4.36 × 10^3^/particle for HPV01 peptide loading and 9.34 × 10^2^/particle for the HPV08 peptide loading. For consistency, the matching amount of each antigens across each experimental groups were used for immunogenicity studies.

#### Antigen Specific Immunogenicity Induced by HPV(peptide)-PSNPs Nanovaccine Formulations

**Figure 2:**
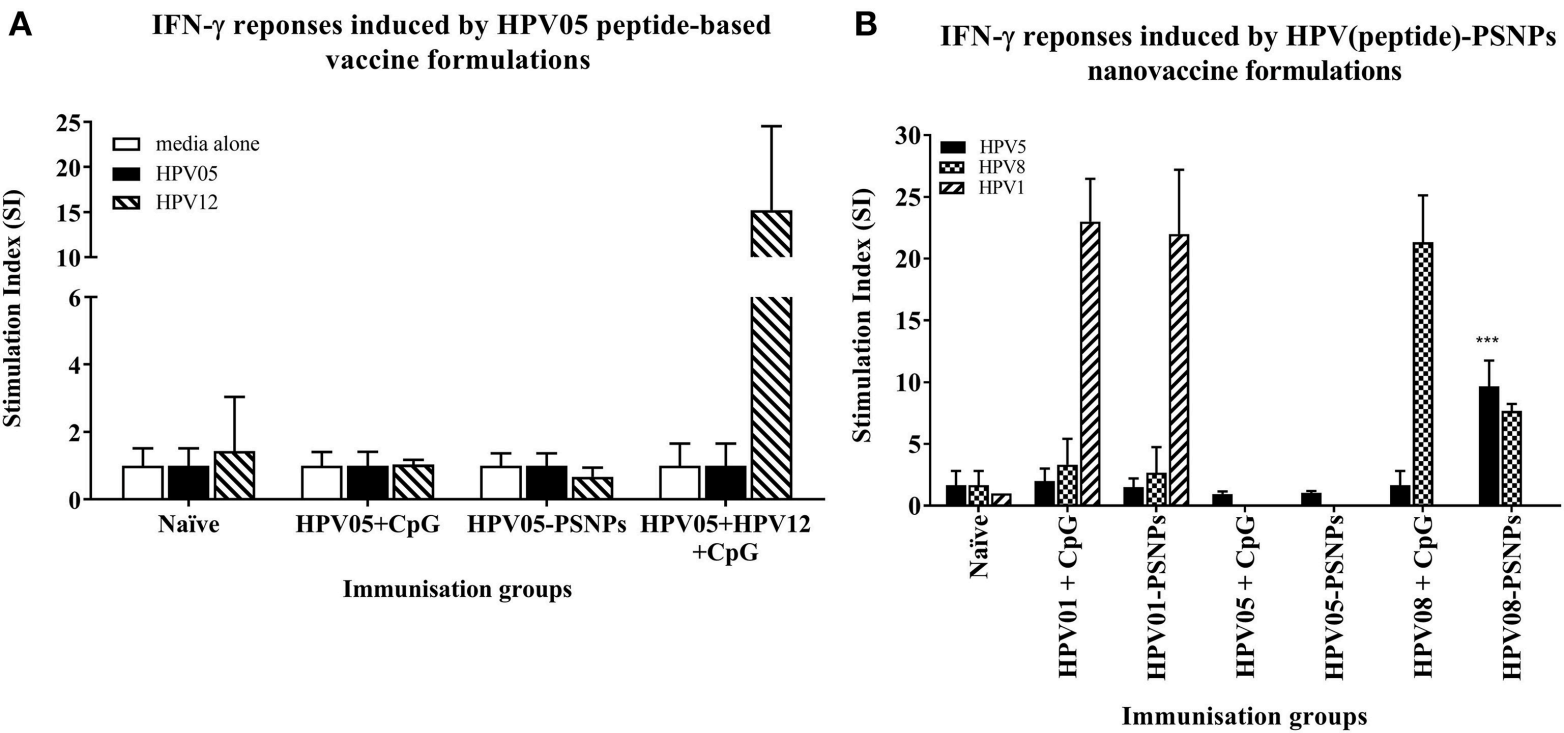
Antigen-specific T cell responses in HLA-A2.1/Kb mice induced by HPV peptides with CpG or PSNPs. HPV01, -HPV05 and –HPV08 peptides were either mixed with CpG or covalently conjugated to PSNPs forming nanovaccine formulations. Each formulation was injected with matching amount of target peptide antigen (all contained 0.5 μg/peptide antigen/injection in 100–200 μl volume). Matching amount of HPV01, HPV05 and HPV08 peptides were also mixed with CpG (20 μg/injection) as comparison. Mice were immunized once intradermally. 15 days after the immunization, antigen specific T cell responses were evaluated by IFN-γ ELISpot assay upon stimulations with different concentration of antigen specific peptides (5, 10, 20, and 50 μg/ml) or controls (media alone, or Con A). Each condition was tested in triplicate on splenocytes from pooled cells within each group of mice (*n* = 3). Results were expressed as Stimulation Index (SI) of the antigen-induced IFN-γ responses (measured by SFU) over the background levels (media alone responses) (± SD triplicated in assay) upon stimulation with HPV05, HPV08 and HPV01 peptide at 20 μg/ml. ^***^p < 0.001 **(A):** HPV05-PSNPs formulation vs. HPV05+CpG ± HPV12 formulations (representative 1 of 3 experiments); **(B):** HPV01-, HPV05-, and HPV08-PSNPs formulations vs. each peptide adjuvanted by CpG formulations (summarized from multiple experiments) in comparison.

HPV peptide-based nanovaccine formulations HPV01-PSNPs, HPV05-PSNPs or HPV08-PSNPs were injected into different groups of HLA-A2.1/Kb transgenic mice (i.d. at the base of tail), to evaluate their immunogenicity. The HPV HLA-A2.1-restricted minimal CD8^+^ T cell epitope HPV05 (HPV16-E7_86−93_, TLGIVCPI) peptides alone was the first to be tested for their capacity to induce antigen specific CD8^+^ T cell responses in HLA-A2.1/Kb mice, when directly conjugated to PSNPs, or when mixed together with CpG with/without the additional peptide from a CD4^+^ T cell epitope (HPV12). This peptide was selected as it has the predicted capacity to induce MHC class II restricted immunity in either mice or humans (Table 1). Results showed that after one immunization, HPV05 either mixed with CpG or conjugated to PSNPs alone, did not induce a HPV05 antigen specific CD8^+^ T cell response (Figure 2A). Upon mixing with the addition of a CD4^+^ T cell helper epitope (HPV12), high IFN-γ production was observed to the CD4^+^ T cell peptide epitope HPV12 itself, but no CD8^+^ T cell response could be elicited (Figure 2A). These results indicated that the HPV minimal CD8^+^ T cell epitope alone, or with added CD4^+^ T cell help, was not capable of provoking an antigen specific CD8^+^ T cell response.

HPV01 (consisting of HPV16-E7_82−94_ and HPV16-E6_41−65_) and HPV08 (HPV16-E7_69−93_) are long peptide antigens which both include the CD8^+^ T cell epitope HPV05 (HPV16-E7_86−93_), but in a different surrounding amino acid context, by including different CD4^+^ T epitopes into their sequence (Table 2). Nanovaccine formulations with either of these two peptides conjugated to PSNPs were used to immunize animals (mice). Antigen specific response to the HPV16-E7_86−93_ HLA-A2.1-restricted CD8^+^ T cell epitope (HPV05) were observed upon HPV08-PSNPs, but not HPV01-PSNPs vaccination in HLA-A2.1/H2Kb transgenic mice, even after one immunization (Figure 2B), indicating that the minimal HLA-A2.1-restricted CD8^+^ T cell epitope (TLGIVCPI) contained in HPV08 was efficiently processed and presented on HLA-A2.1 molecules. By contrast, the formulations with CpG for either of these two peptides (HPV01 and HPV08) did not elicit a CD8^+^ T cell TLGIVCPI-specific responses, despite being generally immunogenic as full-length sequences (Figure 2B). These data suggest differences in antigen processing by CpG and nanovaccines for CD8^+^ T cell epitopes, which in this case have identified HPV08 as a suitable peptide target to be used for the development a peptide based nanovaccine to elicit HPV05 responses against cancers induced by HPV16-E7.

#### Optimization of Immunization Schedules

**Figure 3:**
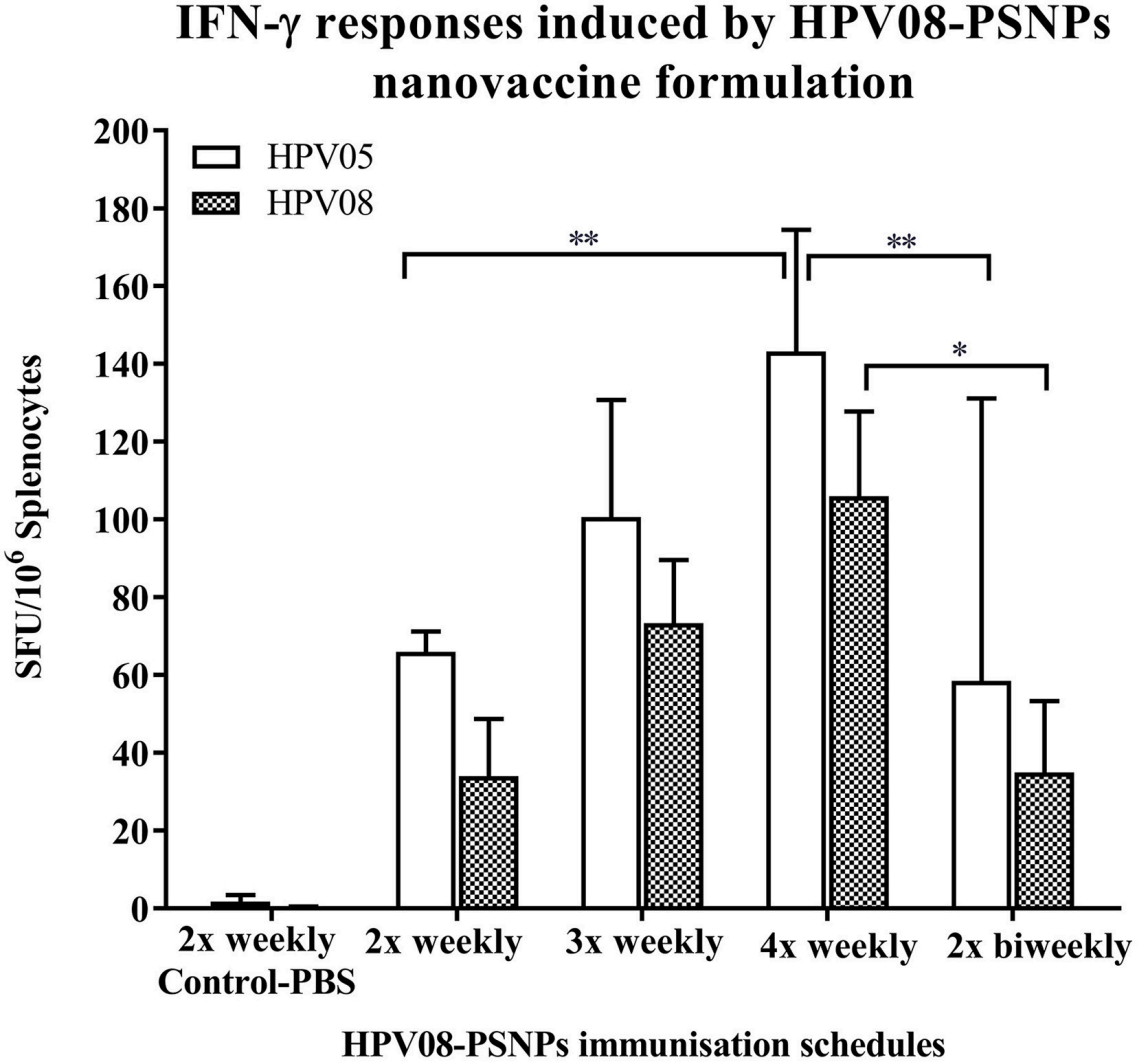
Impact of immunization schedules and time interval on HPV08-PSNPs immunogenicity. HPV08 peptides were covalently conjugated to PSNPs forming HPV08-PSNPs nanovaccine formulation (final containing 0.37 mg/ml of HPV08 conjugated to PSNPs, 100 μl (or 37 μg)/injection). Mice were immunized following the schedules listed in the figure. Twelve days after the last immunization, antigen specific T cell responses were evaluated by IFN-γ ELISpot assay upon stimulations with antigen specific peptides (HPV05 and HPV08, all at 25 μg/ml) or controls (media alone, or Con A). Each condition was tested in triplicate on splenocytes from individual mouse (*n* = 4). Results are expressed as net spot-forming-unit (SFU)/million splenocytes/mouse upon each peptide recall ± SD (*n* = 4 individual mice). Two-way ANOVA analysis indicated the significance of HPV05 and HPV08 peptides induced specific responses in the HPV08-PSNPs formulations ^*^p < 0.05, ^**^p < 0.01.

We further explored the potential for changes in immunization schedule to improve the potency of the HPV08-PSNPs nanovaccine formulation. Specifically, we assessed the impact of changing the time interval between each immunization (Figure 3). The HLA-A2.1 transgenic mice were injected with the same batch of HPV08-PSNPs (i.d. at the base of tail) following the schedules of 2x-weekly, 3x-weekly, 4x-weekly and 2x-biweekly. The overall levels of the immune responses to the native HLA-A2 epitope (HPV05) and to the immunogen itself (HPV08) were generally increased with each additional immunisations scheduled from 2x to 4x weekly immunisations (Figure 3); although the 2x-weekly immunisations were also similar to the 2x-biweekly injections in the overall induction of HPV05 and HPV08 immune responses. The 2x-weekly immunization schedules produced more consistent levels (less “mouse-to-mouse” variability) of the immune responses to HPV05 than the 2x-biweekly immunization schedules. This clearly showed that shortening the time between immunizations to 7 days was not detrimental for CD8^+^ T cell immune response induction upon HPV-PSNPs vaccination (no T cell response exhaustion) and might even be beneficial. Therefore, intradermal immunization with HPV08-PSNPs induced antigen-specific IFN-γ responses against the minimal HLA-A2.1-restricted CD8^+^ T cell epitopes HPV05 in HLA-A2.1/Kb transgenic mice. Increasing number of immunisations positively increased the overall immune responses with the strongest immune response observed after 4x weekly immunizations.

### WT1 Peptide-Based Nanovaccine Formulations and Immunogenicity

#### WT1 Peptide Antigen Design and Selection

The Wilms' tumor antigen 1 (WT1) has been shown to be highly expressed and plays an oncologic role in various hematological and solid malignancies (51), but is negligibly expressed in normal tissues, thus making WT1 an ideal target for cancer immunotherapy strategies (52). WT1 has been listed among the top of the 75 ideal cancer antigens in immunotherapies by the U.S. National Cancer Institute (37). In humans, peptide-based vaccines with HLA-A24-restricted WT1_235−243_ epitopes have been well characterized in the literature to elicit WT1-specific CD8^+^ T cell responses in adult and children cancer patients with the HLA-A24 allele (52–56). Although the CD8^+^ T cell responses toward the HLA-A2.1-restricted WT1_126−134_ epitope “RMFPNAPYL” (herein called WT1A, Table 4) have been identified in various HLA-A2^+^ cancer patients, research and clinical trials using WT1A peptide vaccination strategies have been disappointing (57, 59, 60). The WT1A-specific CD8^+^ T cell responses were either short-lived with repeated vaccinations enriching for lower avidity populations (59) or could not be further expanded *in vitr*o and may have been functionally impaired following WT1A vaccination (60). A modified version to substitute an arginine (R) to tyrosine (Y) at position 1 (***Y***MFPNAPYL, herein called WT1B, Table 4) has been shown to increase the peptide binding and stability to the HLA-A2.1 molecule (58). WT1B has been shown to be recognized by the native WT1A in humans (58). Our previous studies (61) also demonstrated that both WT1A and WT1B vaccination (adjuvanted by CpG) generated functionally similar CD8^+^ T cell responses to the cognate antigen *ex vivo*, and both vaccination regimens could be readily expanded in response to the cognate peptide. While WT1A generated greater WT1A-specific CD8^+^ T cell responses, WT1B showed greater potential to generate a proportion of dual responses that cross-reacted with WT1A, and could be expanded by the WT1A peptide (61). To further potentially promote better responses to WT1B (that would further be able to cross-react with the native epitope WT1A), based on our findings with HPV05 and HPV08, we designed variant peptides which could contain WT1B within an extended peptide (WT1C, WT1D, and WT1E, Table 3), conjugated them to the PSNPs to form WT1 peptide-PSNPs nanovaccine formulations, and evaluated their ability at inducing antigen specific CD8^+^ T cell responses. In this case, we also extended the sequence at both the carboxy and amino ends with what would have been the native WT1A context (WT1C). Additionally, we followed recent literature suggesting that flanking amino acids with aromatic (tyrosine, Y), basic (lysine, K), and small aliphatic side chains (alanine, A) supported efficient cytotoxic T lymphocyte (CTL) recognition epitopes (62), and an additional AAY amino acid sequence was included at the amino end of WT1B to generate the WT1D peptide in the attempt to increase the CD8^+^ T cell epitopes processing and recognition. To further explore providing processing context to both side of the epitopes, we generated WT1E, which is WT1D plus the same extension at the carboxy end as WT1C (Table 4).

**Table 4:** WT1 peptide antigens (the predicted CD8^+^ T cell epitopes are underlined).

**Peptide code**	**Sequence**	**Amino acid position**	**Function**
WT1A	**R**MFPNAPYL	WT1_126−134_	Minimal native HLA-A2-restricted CD8^+^ epitope (57)
WT1B	**Y**MFPNAPYL	WT1_126−134_	WT1A variant with higher binding affinity. One amino acid substitution at position 1 by tyrosine (Y) instead of arginine (R) (58).
WT1C	SGQAYMFPNAPYLPSCLES	WT1_122−140_	Consisting both CD8^+^ (WT1_126−134_, HLA-A2-restricted) and CD4^+^ (WT1_124−138_, HLA-DRB1 and DR15, DR53-restricted) epitopes
WT1D	AAYYMFPNAPYL	AAY+ WT1_126−134_	Modified WT1B sequence including an extended sequence (AAY) at flanking region to increase epitope recognition, still consisting of WT1_127−134_, an HLA-A2-restricted CD8^+^ epitope
WT1E	AAYYMFPNAPYLPSCLES	AAY+ WT1_126−140_	Modified WT1D sequence with additional sequence for CD4^+^ epitope at C-terminal; consisting of both HLA-A2-restricted CD8^+^ epitope and CD4^+^ epitope (HLA-DRB1, -DR15 and DR53-restricted)

#### Covalently Linking the WT1 Peptide Candidates to Nanoparticles (PSNPs) and Optimization of the Peptide-PSNPs Formulations

**Figure 4:**
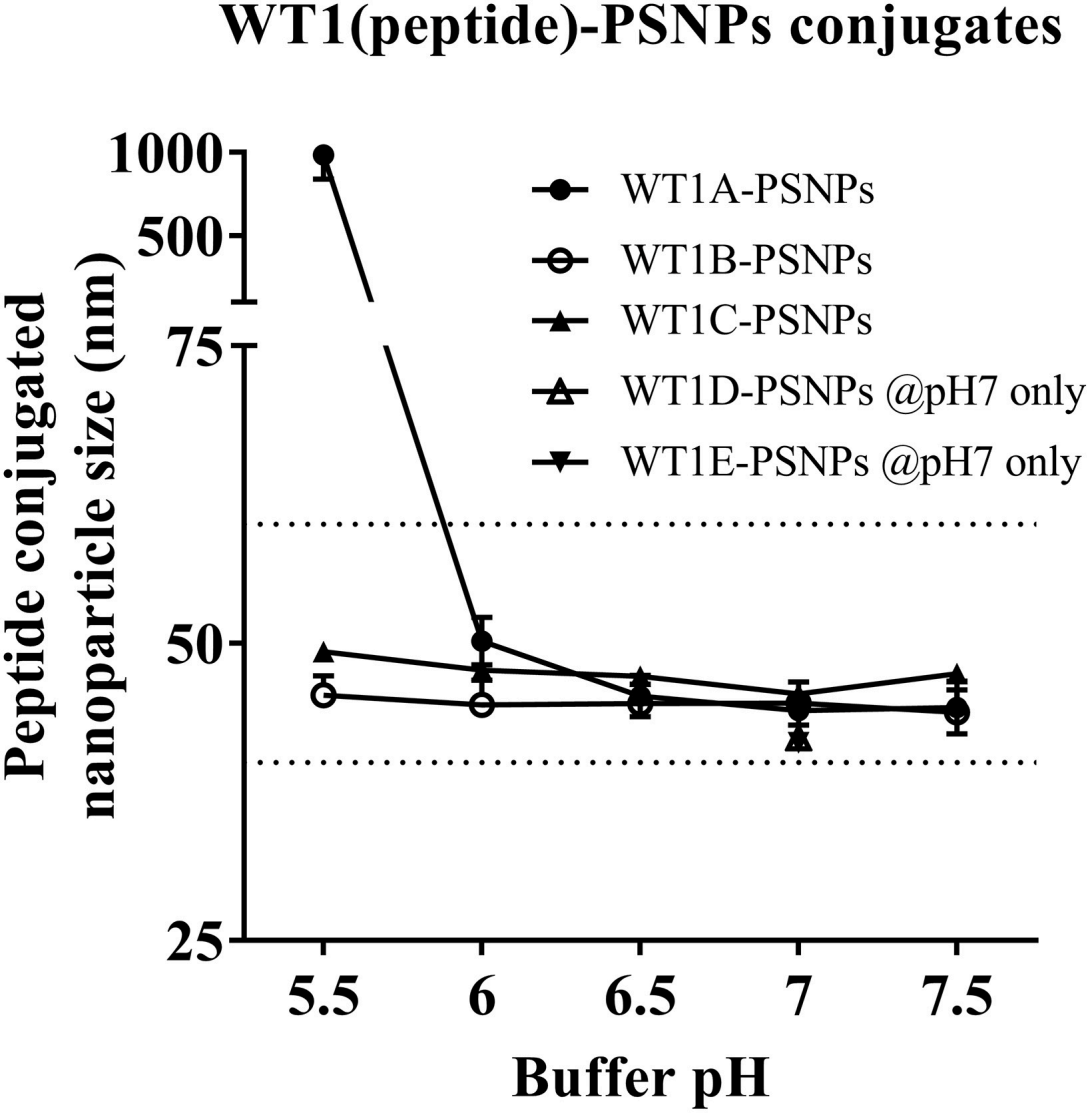
Optimization of conjugation conditions to covalently conjugate WT1 peptides to PSNPs to produce uniform WT1(peptide)-PSNPs nanovaccine formulations. PSNPs (1% solid final) were pre-activated following the standard procedure (detailed in Materials and Methods), and then re-conditioned in different buffer and pH solutions before mixing with each peptide antigen (0.5 mg/ml final) for conjugation. After conjugation, the final particle sizes for each peptide-PSNPs formulation was assessed using a Zetasizer. Data presented as peptide-PSNPs conjugate size (nm) ± SD (3 repeated measurements) under each conjugation conditions for each peptide. The dotted lines indicated the acceptable nanovaccine formulation size range at 40–60 nm.

Conjugations of WT1 peptides to the PSNPs were tested in PBS at the various pH ranges. As shown in Figure 4, WT1A and WT1B peptides were conjugated over a range of pH conditions in PBS during the conjugation step, WT1A-PSNPs formulation aggregated in pH=5.5 buffer condition, but were stable when pH>6; whereas WT1B-PSNPs formulation were stable and no aggregation was observed over the pH ranges tested. Therefore, the optimal pH range for all WT1 peptides candidates was 6.5–7.5. All other WT1 peptides (WT1C, WT1D, and WT1E) were conjugated to PSNPs at pH 7.1, and final conjugated nanovaccine formulations were uniform in sizes (ranging between 40 and 60 nm, with *Pdl* <0.2). Table 5 summarizes the optimal conjugation conditions for each of the WT1 peptide candidates evaluated in the study. The overall conjugation efficiency was excellent (up to 100% by HPLC analysis), and antigen loadings (number of peptide molecules/particle) were also high (Table 5). For consistency, the matching amount of each antigens across each experimental groups were used for immunogenicity studies.

**Table 5:** Optimal conjugation conditions for the WT1(peptide)-PSNPs formulations.

**Peptide-PSNPs**	**Buffer**	**pH**	**Size (nm)**	**Polydispersity (Pdl)**	**Conjugation efficiency (%)**	**Antigen loading (peptide molecules/particle)**
WT1A-PSNPs	PBS	7.1	44.67 ± 0.45	0.09 ± 0.01	100^*^	2.24 × 10^3^
WT1B-PSNPs	PBS	7.1	47.11 ± 1.42	0.10 ± 0.02	100^*^	1.41 × 10^3^
WT1C-PSNPs	PBS	7.1	45.80 ± 2.17	0.07 ± 0.02	100^*^	1.61 × 10^3^
WT1D-PSNPs	PBS	7.1	42.00 ± 0.19	0.05 ± 0.00	44^#^	7.51 × 10^2^
WT1E-PSNPs	PBS	7.1	41.66 ± 0.45	0.05 ± 0.00	60^#^	7.12 × 10^2^

* Conjugation efficiency determined by HPLC amino acid analysis.

# *conjugation efficiency determined by BCA assay. The overall conjugation efficiencies were low, and this was due to the specific amino acid contents interfering with the BCA assay, subsequently also impacting the calculation for the antigen loading/particle*.

#### Antigen Specific CD8^+^ T Cell Responses Induced by WT1(peptide)-PSNPs Nanovaccine Formulations

**Figure 5:**
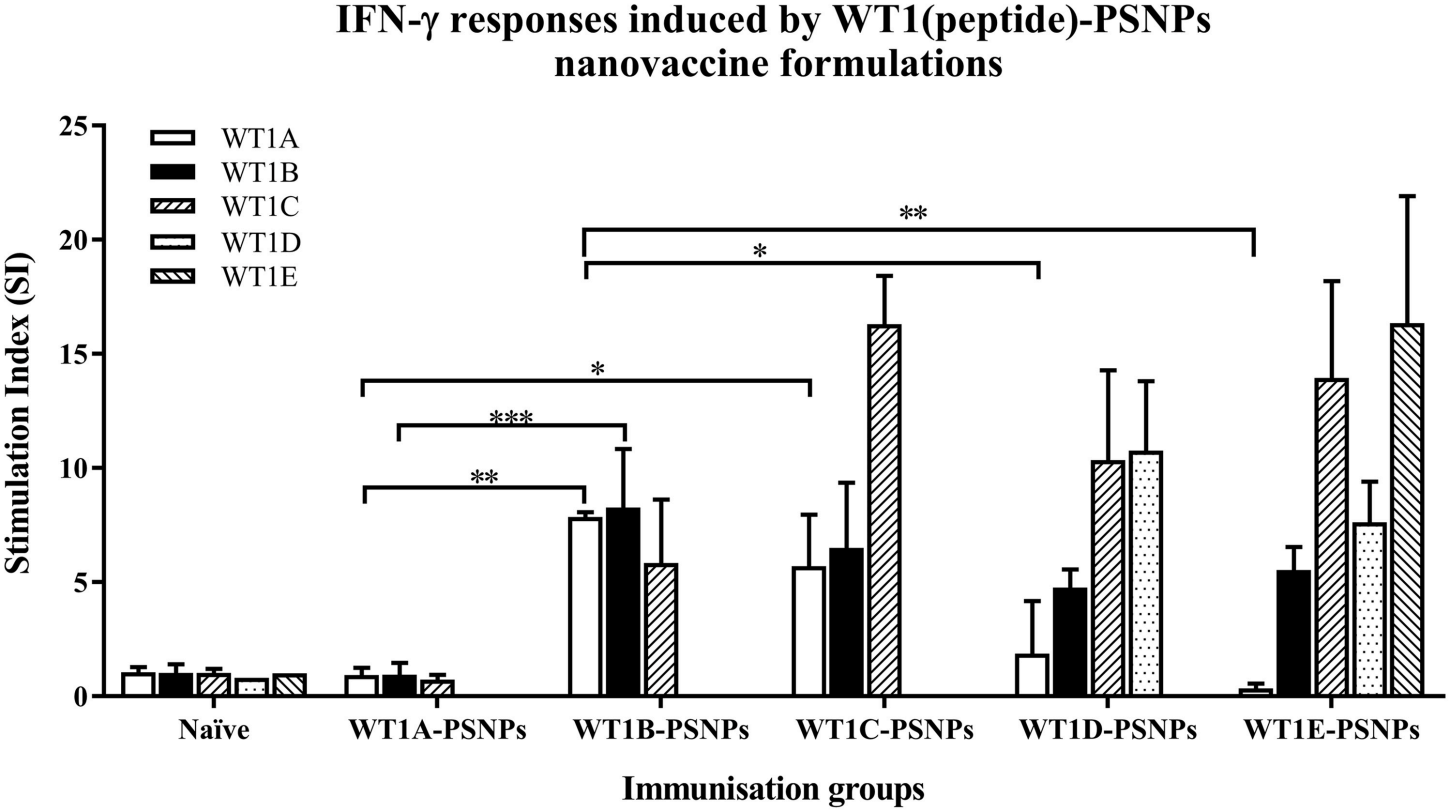
Induction of IFNγ-producing antigen specific CD8^+^ T cells following i.d. administrations of WT1(peptide)-PSNPs candidates in HLA-A2.1/Kb mice. WT1 derived peptides (WT1A, WT1B, WT1C, WT1D, and WT1E) were covalently conjugated to PSNPs to constitute PSNPs vaccine formulations (containing 0.5 mg/ml of each peptide in each of the conjugation mix). Mice were immunized 3 times with each formulation (100 μl or 50 μg (including both conjugated and non-conjugated peptide)/injection) intradermally, 10 days apart. 11 days after the last immunization, antigen specific T cell responses were evaluated by IFN γ ELISpot assay upon stimulations with WT1 peptides (5 μg/ml) or controls (media alone or Con A). Each condition was tested in triplicate on splenocytes from individual mouse (*n* = 4). Results are expressed as stimulation index (SI) of the SFU over the background (media alone) ± SD (*n* = 4 individual mice). Two-way ANOVA analysis indicated the significance of WT1A and WT1B peptide processing in the WT1peptide-PSNPs formulations. ^*^p < 0.05, ^**^p < 0.01; ^***^p < 0.001. Figure was summarized from multiple experiments.

The WT1 peptide-based nanovaccine formulations (WT1A-PSNPs, WT1B-PSNPs, WT1C-PSNPs, WT1D-PSNPs, and WT1E-PSNPs) were injected into HLA-A2.1/Kb transgenic mice (i.d. at the base of tail) to evaluate their immunogenicity (see material and methods section and figure legends for details). Results in Figure 5 show that intradermal immunization with WT1B-, WT1C-, or WT1D-PSNPs formulations, but not with WT1A-PSNPs, induced antigen-specific IFN-γ responses to the HLA-A2.1-restricted CD8^+^ T cell epitopes WT1A (RMFPNAPYL, native sequence) and its variant WT1B (***Y***MFPNAPYL) (^**^p < 0.01, ^*^p < 0.05, ^*^p < 0.05, respectively). Despite the fact that the WT1C-PSNPs formulation contained both CD8^+^ and CD4^+^ T cell epitopes, there were negligible differences in the CD8^+^ T cell specific responses elicited, between the two formulations, although there was a trend for a better induction of antigen-specific T cell responses to the native epitope WT1A in WT1B-PSNPs vaccinated animals. Additional of the amino acid sequence (AAY) at the flanking region of the WT1B peptide has been reported to promote appropriate processing and recognition of the minimal epitope (62), but this was not observed in our study, as the incorporation of this sequence did not enhance responses to the minimal epitope WT1B, and even decreased the cross-reactive CD8^+^ T cell responses to the native WT1A antigen, when comparing WT1D-PSNPs and WT1E-PSNPs induced responses to the other formulations (^*^p < 0.05 and ^**^p < 0.01, respectively) (Figure 5). Therefore, in the case of WT1 peptide antigen, substituting an amino acid [arginine (R) to tyrosine (Y)] generated strong immune responses to itself as well as cross-reactive responses to the native WT1A epitope, but extending the minimal CD8^+^ T cell epitope by incorporating amino acids derived from its natural context, or predicted to potentially promote processing, did not enhance the CD8^+^ T cell immune responses being induced.

### Survivin Peptide-Based Nanovaccine Formulations and Its Immunogenicity

#### Survivin Peptide Antigen Design

Survivin (SV) is an oncogenic inhibitor-of-apoptosis protein (142 aa) crucial for the survival of tumor cells. It is generally expressed at low to negligible levels in normal tissue but is over expressed in a wide variety of cancers including lung, breast, pancreatic, colorectal, stomach and ovarian tumors as well as hematological malignancies (63). It is the fourth most highly expressed transcript in human cancer cells (26), and has been found to be over-expressed in up to 90% of ovarian cancers (64, 65), making it potentially a good target for vaccine based treatment for ovarian cancer. However, despite the fact that Survivin peptides have been studied in multiple clinical trials, confirming their safety (66, 67), Survivin has been only weakly immunogenic, and hence not protective, across most studies (63, 68). A different choice of antigen delivery and adjuvant system could potentially enhance the immunogenicity of this protein. Both CD4^+^ and CD8^+^ T cells epitopes from Survivin protein are important for induction of effective anti-tumor immune response (63). Given the PSNP nanoparticle vaccine approach has been successful in delivering peptide antigens [see above and previous publications (13, 69)], we explored how to increase the immunogenicity of a lead Survivin peptide containing CD8^+^ T cell epitope, using these nanoparticle formulations. A number of Survivin-derived candidate peptides were identified based on an extensive literature search and clinical trials (70–73) and manufacturing feasibility (Table 6). The HLA-A2.1 restricted CD8^+^ T cell native epitope peptide SV03 (SV_95−104_) and SV04 (SV_96−104_) were mostly cited by literature (70, 71, 75–78). In order to increase the minimal CD8^+^ T cell epitope binding affinity to the HLA-A2.1 allele and subsequently to increase the immune responses, modified versions of SV03 and SV04 peptides were made by substituting the amino acid Threonine (T) to Methionine (M) at the position 97 (EL***M***LGEFLKL, herein named SV11 and SV10) as an agonist for use with PSNP vaccines. To further potentially encourage appropriate antigen processing and the epitope recognition to the HLA-A2.1 molecule, “AAY” amino acid sequence at the amino flanking region of the SV10 was also included (*AAY*LMLGEFLKL, named SV16). Additional panel of peptides were also designed to incorporate both CD8^+^ and CD4^+^ T cell epitopes (for potential downstream use in humans) in the peptide antigen sequences and evaluated for immunogenicity in PSNPs nanovaccine formulations in this study, such as SV01 (SV_90−124_), SV02 (SV_2−36_), and SV12 (Table 6). SV01 and SV02 contained both CD8^+^ and CD4^+^ T cell epitopes. SV01 (SV_90−124_) covers multiple HLA-A2.1 and HLA-A1-restricted CD8^+^ T cell epitopes (SV_92−101_, _95−104_) (70, 79), as well as HLA-DR1, DR3, DR4-restricted CD4^+^ T cell epitopes (SV_97−111, 110−124_) (72, 73), good coverage for both MHCI and MHC II recognition. SV02 (SV_2−36_) contains HLA-A2.1-restricted CD8^+^ T cell epitopes (SV_5−14, 18−28_) (68, 70) and promiscuous HLA-DR-restricted (HLA-DR1, 15, 3,7,13,11) CD4^+^ T cell epitopes (SV_10−24, 22−36_) (72). SV12 (SV_53−67_ variant: M57) contains multiple CD8^+^ T cell epitopes (cross-reactive to both H2Kb and HLA-A2) and promiscuous HLA-DR-restricted CD4^+^ T cell epitopes (68).

**Table 6:** Survivin peptide antigens (the predicted CD8^+^ T cell epitopes are underlined).

**Peptide code**	**Sequence**	**Amino Acid position**	**Function**
**PEPTIDES SELECTED AS ANTIGEN TARGETS FOR NANOVACCINES**			
SV01	KKQFEELTLGEFLKLDRERAKNKIA KETNNKKKEF	SV_90−124_	Containing both HLA-A1 and A2.1 restricted CD8^+^ (SV_92−101_, _95−104_) and HLA-DR1, 3, 4-restricted CD4^+^ T cell epitopes (SV_97−111, 110−124_)
SV02	GAPTLPPAWQPFLKDHRISTFKNWPFL EGCACTPE	SV_2−36_	Containing both HLA-A2.1-restricted CD8^+^ (SV_5−14, 18−28_) and HLA-DR1, 15, 3,7,13,11-restricted CD4^+^ T cell epitopes (SV_10−24, 22−36_)
SV10	L**M**LGEFLKL	SV_96−104_ variant	As above, SV_97_: T to M
SV12	DLAQ**M**FFCFKELEGW	SV_53−67_ variant (SV_57_: M)	Containing multiple CD8^+^ T cell epitopes (cross-reactive to both H2Kb and HLA-A2) and promiscuous HLA-DR-restricted CD4^+^ T cell epitopes (68)
SV16	AAYLMLGEFLKL	AAY+SV10 (SV_96−104_ variant)	As above, SV_97_: T to M
**PEPTIDES FOR RECALL ANTIGEN SPECIFIC REACTIVITY IN ELISpot ASSAY:**			
SV03	ELTLGEFLKL	SV_95−104_	HLA-A2.1-restricted CD8^+^ T cell epitope (70)
SV04	LTLGEFLKL	SV_96−104_	HLA-A2.1-restricted CD8^+^ T cell epitope (71)
SV05	TLPPAWQPFL	SV_5−14_	HLA-A2.1-restricted CD8^+^ T cell epitope (70)
SV06	RISTFKNWPFL	SV_18−28_	HLA-A2.1-restricted CD8^+^ T cell epitope (68)
SV07	LTLGEFLKLDRERAKN	SV_96−111_	HLA-DR1, DR3, DR4-restricted CD4^+^ T cell epitopes (73)
SV08	WQPFLKDHRISTFKN	SV_10−24_	Promiscuous HLA-DR1, DR15, DR3, DR7, DR13, DR11-restricted CD4^+^ epitopes (72)
SV09	HRISTFKNWPFLEGCACT	SV_17−34_	CD4^+^ T cell epitope (74)
SV11	ELMLGEFLKL	SV_95−104_ (SV03) variant	SV_97_: T to M, consists CD8^+^ T cell epitope
SV13	KKQFEELMLGEFLKL	SV_90−104_ variant	SV_97_: T to M, consists CD8^+^ T cell epitope (extended SV11)
SV14	KKQFEELMLGEFLKLDRERAK	SV_90−110_ variant	SV_97_: T to M, consists both CD8^+^ and CD4^+^ T cell epitopes (SV07)

#### Covalently Linking the Survivin Peptide Candidates to Nanoparticles (PSNPs) and Optimization of SV(peptide)-PSNPs Nanovaccine Formulations

**Figure 6:**
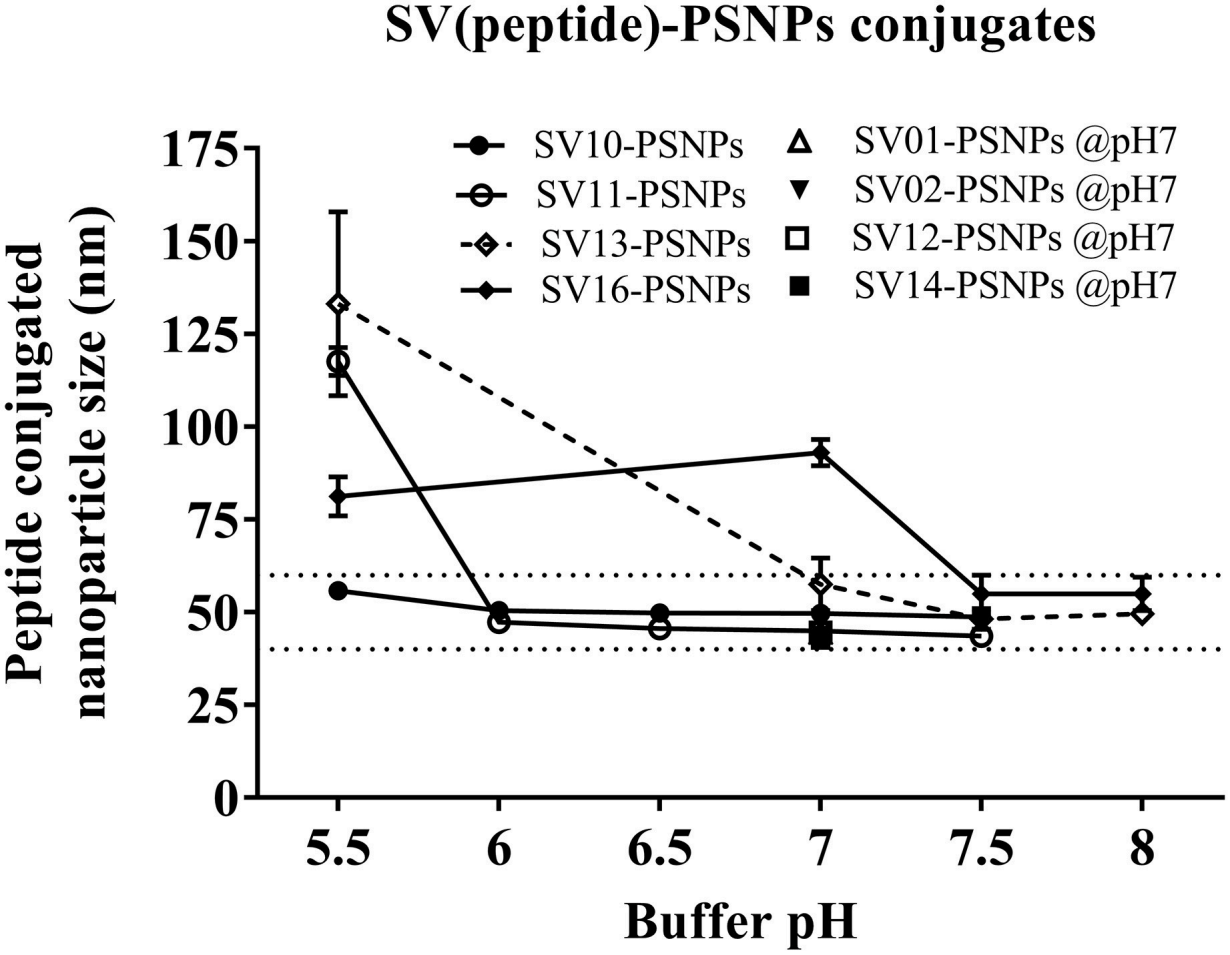
Optimization of conjugation conditions to covalently conjugate Survivin peptides to PSNPs to produce uniform SV(peptide)-PSNPs nanovaccine formulations. PSNPs (1% solid final) were pre-activated following the standard procedure (detailed in Materials and Methods), and then re-conditioned in different buffer and pH solutions before mixing with each peptide antigen (0.5 mg/ml final) for conjugation. After conjugation, the final particle sizes for each peptide-PSNPs formulation was assessed using a Zetasizer. Data presented as peptide-PSNPs conjugate size (nm) ± SD (3 repeated measurements) under each conjugation conditions for each peptide. The dotted lines indicated the acceptable nanovaccine formulation size range at 40–60 nm.

Conjugations of Survivin peptides to the PSNPs were tested in PBS at the various pH. As results shown in Figure 6, SV10, SV11, SV13, and SV16 peptides were conjugated over a range of pH conditions in PBS during the conjugation step, apart from SV10, the SV11-, SV13-, and SV16-PSNPs formulations aggregated at pH=5.5 buffer condition and aggregations were reduced with the increasing pH, optimal at pH 7–8. The SV10-PSNPs formulation were stable and there was no aggregation over the pH ranges tested. Therefore, the optimal pH range for all SV peptides candidates were 7–7.5. All other SV peptides (SV01, SV02, SV12, and SV14) were conjugated to PSNPs at pH 7.1, and final conjugated nanovaccine formulations were uniform in sizes (range between 40 and 60 nm, with *Pdl* <0.2). Table 7 below summarizes the optimal conjugation conditions for each of the SV peptide candidates evaluated in this study. All SV peptides were able to be conjugated to the PSNPs with high conjugation efficiency, and ultimately high levels of antigen loading represented by the number of peptide molecules per particle (Table 7). For consistency, the matching amount of each antigens across each experimental groups were used for immunogenicity studies.

**Table 7:** Optimal conjugation conditions for the SV(peptide)-PSNPs formulations.

**Peptide-PSNPs**	**Buffer**	**pH**	**Size (nm)**	**Polydispersity (Pdl)**	**Conjugation efficiency (%)**	**Antigen loading (peptide molecules/particle)**
SV01-PSNPs	PBS	7.1	44.48 ± 0.12	0.1 ± 0.01	85.4^#^	6.07 × 10^2^
SV02-PSNPs	PBS	7.1	43.68 ± 0.52	0.06 ± 0.00	87.8^#^	5.67 × 10^2^
SV10-PSNPs	PBS	7.1	45.94 ± 0.88	0.17 ± 0.02	64.7^*^	1.56 × 10^3^
SV11-PSNPs	PBS	7.1	44.96 ± 0.61	0.09 ± 0.01	ND	-
SV12-PSNPs	PBS	7.1	42.37 ± 0.22	0.09 ± 0.00	64.2	8.33 × 10^2^
SV13-PSNPs	PBS	7.1	43.15 ± 0.14	0.09 ± 0.01	ND	-
SV14-PSNPs	PBS	7.1	42.49 ± 0.13	0.08 ± 0.00	ND	-
SV16-PSNPs	PBS	7.5	43.48 ± 0.14	0.09 ± 0.01	86.91^#^	1.54 × 10^3^

* *Conjugation efficiency determined by HPLC amino acid analysis*.

# *conjugation efficiency determined by BCA assay*.

*ND: not determined due to the specific amino acid content interfering with the BCA assay*.

#### Antigen Specific Immunogenicity Induced by SV(peptide)-PSNPs Nanovaccine Formulations

**Figure 7:**
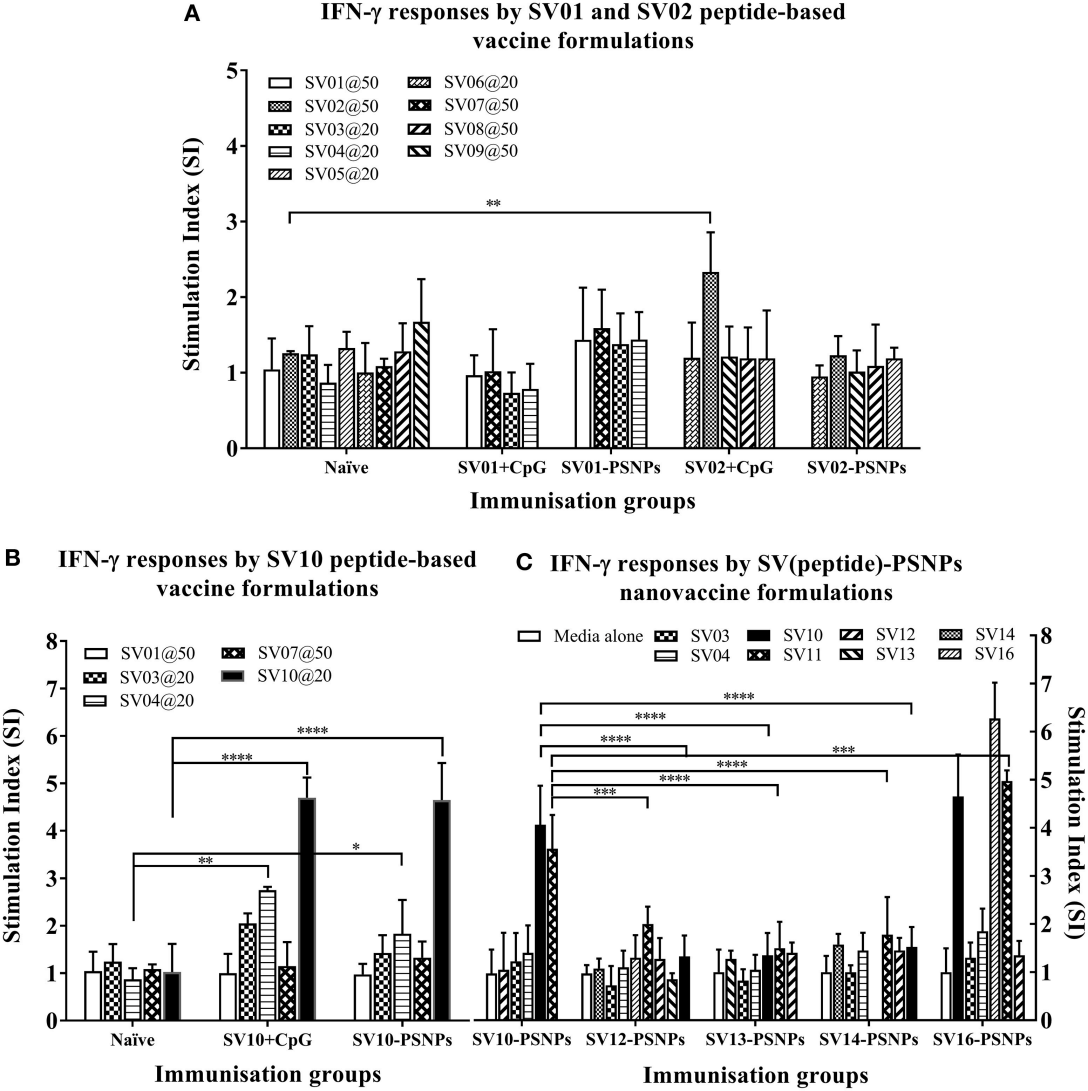
Antigen-specific T cell responses in HLA-A2.1/Kb mice induced by SV peptides with CpG or PSNPs. SV-derived peptides: **(A)** SV01 and SV02, **(B)** SV10, **(C)** SV10, SV12, SV13, SV14, and SV16 were covalently conjugated to PSNPs forming PSNPs vaccine formulations. Each formulation contained equal amount of each SV peptide target and PSNPs (all at 0.5 mg/ml per peptide, 1% solid for PSNPs; 100 μl/injection). Equivalent amount of SV01, SV02, and SV10 peptides were also mixed with CpG (20 μg/injection) as comparison. For each immunization group, mice were immunized 3 times intradermally, 10 days apart. 11 days after the last immunization, antigen specific T cell responses were evaluated by IFN-γ ELISpot assay upon stimulations with antigen specific peptides (dosages on the figure (μg/ml) except C all at 25 μg/ml) or controls (media alone, or Con A). Each condition was tested in triplicate on splenocytes from individual mouse (*n* = 3–4). Results are expressed as the Stimulation Index (SI) of the antigen-induced IFN-γ responses (measured by SFU) over the background levels (media alone responses) ± SD (*n* = 4 individual mice) upon stimulation for each peptide conditions assayed in triplicated wells. Two-way ANOVA analysis indicated the significance of antigen specific responses induced by specific peptides in the SVpeptide-PSNPs or SVpeptide+CpG formulations. ^*^p < 0.05, ^**^p < 0.01; ^***^p < 0.001, and ^****^p < 0.0001. Figure was summarized from multiple experiments.

The Survivin peptide-based nanovaccine formulations were injected into HLA-A2.1/Kb transgenic mice (i.d. at the base of tail) to evaluate their immunogenicity (see material and methods section and figure legends for details). The long 35aa peptides SV01 (SV_90−124_) and SV02 (SV_2−36_) which contain multiple CD8^+^ and CD4^+^ T cell epitopes as well as SV10 (minimal CD8^+^ T cell epitope SV_96−104_ variant,), were the first to be evaluated in the PSNPs conjugated nanovaccine formulations. Results in Figure 7A, showed that when SV01 peptides were conjugated to PSNPs or mixed with CpG and tested for antigen specific immune responses against the recall peptides SV03, SV04, SV07, or itself (SV01), none of them induced antigen specific IFN-γ T cell responses. When SV02 peptides were conjugated to PSNPs or mixed with CpG, and tested against the recall peptides SV05, SV06, SV08, SV09 or itself (SV02), only the SV02 peptide was able to induce a very weak IFN-γ responses in the SV02+CpG formulation (SI = ~2, ^**^p < 0.01), but not SV02-PSNPs, when compared to the background. Therefore, both SV01 and SV02 peptides were not able to substantial CD8^+^ T cell responses to the native HLA-A2.1 restricted epitopes SV_95−104_, SV_96−104_, SV_5−14_ and SV_18−28_ (SV03, SV04, SV05, and SV06, respectively) either in formulations conjugated to PSNPs or adjuvated by CpG. No CD4^+^ T cell mediated IFN-γ responses observed to any of the other recall CD4^+^ T cell epitopes SV_96−111_, SV_10−24_ and SV_17−34_ (SV07, SV08, and SV09, respectively) (Figure 7A).

However, the SV10 peptide (an agonist L***M***LGEFLKL peptide epitope for the natural epitope SV04 (SV_96−105_) antigen conjugated to PSNPs (SV10-PSNPs) was able to generate strong IFN-γ responses to itself (^****^p < 0.0001, Figure 7B) with responses equivalent to those elicited by the CpG adjuvated SV10 peptide formulation. Meanwhile, very weak but significant responses were also induced to the SV04 peptide in both formulations compared to the naïve group (^**^p < 0.01 and ^*^p < 0.05 for CpG and PSNPs groups, respectively).

Based on the immunogenicity of the SV10 peptide formulations, we further designed Survivin peptides SV12 (SV_53−67_ agonist variant), SV13 (SV_90−104_ agonist variant), SV14 (SV_90−110_ agonist variant) and SV16, an extended sequence (AAY) at flanking region of SV10 to potentially help increase the epitope processing. We then evaluated their immunogenicity when conjugated to PSNPs. As shown in Figure 7C. However, none of these longer peptides (SV12, SV13 and SV14) containing both CD4^+^ and CD8^+^ T cell Survivin derived natural or agonist epitopes were able to induce antigen specific CD8^+^ T cell responses. By contrast, the CD8^+^ T cell epitope variant SV10, and SV16 (which contains SV10) were able to induce the HLA-A2.1 restricted CD8^+^ T cell responses to SV10 and SV11 (a SV03/SV_95−104_ variant) upon immunization with SV10-PSNPs or SV16-PSNPs vaccine formulations (Figure 7C). Disappointingly however, none of the native or agonist formulations were able to induce strong to the natural SV3 and SV4 Survivin CD8^+^ T cell epitopes.

## Discussion

This comprehensive study assessed the impact of minor relative changes in peptide length and sequence for the induction CD8^+^ T cell responses in HLA-A2.1 transgenic mice to antigens relevant to the development of gynecological cancer vaccines, based on the lead vaccine antigens HPVE7, Survivin and WT1. It focused specifically on their potential to be used in nanoparticle-based vaccine formulations such as PSNPs.

The minimal CD8^+^ T cell peptide epitope HPV05 did not elicit significant immunity using a conventional adjuvant (CpG 1826) or when delivered as a conjugate with PSNPs nanoparticle carriers. This result contrasts previous studies using PSNPs to deliver very high affinity minimal CD8^+^ T cell epitopes such as SIINFEKL (from OVA) (12, 13) or SYIPSAEKI (from *Plasmodium berghei* circumsporozoite protein) (18). Differences in antigen loading would not explain this finding, as there was excellent loading and nanoparticle size retention in an immunogenic range comparable to our previous studies. It has been suggested that lower affinity epitopes may be more dependent on CD4^+^ T cell help (80–82). To address whether our observed lack of response was because of lack of CD4^+^ T cell help, we mixed HPV05 with a known HPV derived CD4^+^ T cell helper epitope (HPV12). However, this approach did not facilitate CD8^+^ T cell induction. By contrast, HPV05 specific responses were elicited when the HPV05 sequence was lengthened at the amino end within its natural context to further include a CD4^+^ T cell epitope, and used to formulate nanoparticle based vaccines. To note, this same extended sequence (HPV08), by contrast, when CpG adjuvanted, elicited responses to the full-length peptide, but failed to induce CD8^+^ T cell responses to HPV05. It is likely that delivering this extended peptide conjugated to PSNPs promoted uptake and helped in the intracellular processing by cross-priming DC, specialized for the induction of CD8^+^ T cells. Indeed previous studies with PSNPs have shown uptake by cross-priming CD8^+^ DC (83) as well as TAP dependency for the priming of CD8^+^ T cells to epitopes contained in PSNP-protein conjugated vaccines (12), indicating further the use of alternative intracellular cross-priming processing pathways (84). Furthermore, CD4^+^ T cell responses could also be elicited to HPV08 in naïve T cell priming cultures from human peripheral blood mononuclear cells (PBMC) (unpublished data).

The minimal HLA-A2.1 binding CD8^+^ T cell epitope WT1A from the WT1 protein conjugated to nanoparticles (PSNPs) similarly failed to induce CD8^+^ T cells by itself, but in this case, it was sufficient to generate a high affinity agonist (WT1B) to produce a bioactive vaccine PSNPs conjugate which was able to induce immune responses to WT1B, which were further cross-reactive with WT1A. Such results suggested that mutated antigens derived from described antigens and upon conjugation with nanoparticles can induce higher grade of immunogenicity. Further extending the sequence at either end of WT1B, modeling it on either the natural peptide context for WT1A, or incorporating the sequence AAY at the amino end [described in the literature as being able to promote better antigen processing and recognition (62)], failed to further enhance CD8^+^ T cell responses generated by vaccines including these formulations. In this specific case therefore, the optimal vaccine may be, simply a minimal high affinity agonist CD8^+^ T Cell epitope conjugated directly to the nanoparticle, similarly to our previous studies using malaria high affinity agonist peptides with PSNPs (18). Similarly, initially negative results were observed using the unmodified Survivin derived minimal CD8^+^ T cell epitopes, SV03 and SV04, and extending the peptide length alone and conjugating to PSNPs was not able to rescue CD8^+^ T cell induction. SV02 and SV04 are particularly weak binders to MHC class I (68, 70, 71), and known to be difficult epitopes in that there is a level of endogenous tolerance as self-antigens (85). In this case, we also trialed the testing of a super agonist variant (SV10), which has been used in human clinical trials in the context of other adjuvants, to explore its potential utility in nanoparticle-based formulations. Similarly to what we observed with WT1, using the agonist SV10 coupled directly to the nanoparticles was able to induce substantial CD8^+^ T cell responses to SV10. Disappointingly, these responses were not cross-reactive to the native SV03 and SV04 sequences. Further extending the SV10 sequence within the natural SV03/04 context to generate longer peptides, did not increase or broaden, and even decreased reactivity to SV10 itself. By contrast, adding the AAY sequence at the amino end did result in enhanced immune responses to SV10, but these enhanced responses were not accompanied by a broadening of reactivity to include cross-reactivity with SV03 or SV04. Expanding the spectrum of cross-reactivities may be explored in future studies by further methodically changing the amino acid sequence of SV10 to generate more complex agonists. This approach has been used successfully to expand the spectrum of recognized variant CD8^+^ T cell epitopes in the circumsporozoite protein from *P. berghei* (16) in the context of malaria.

The magnitude of immune responses induced by the formulations in the present study is comparable to our previous studies which have shown tumor protection in diverse animal models [(12, 13, 15) and unpublished]. However, as with any vaccine aiming to induce CD8^+^ T cells, this does not really translate into certainty in obtaining high or tumor protective CD8^+^ T cell responses in humans, as, at best, tumor protection studies in animals, even transgenic animals, can only be indicative of vaccine potential. The aim of this study was not to progress any particular formulation to human trials. If this was an objective in the future it will be important to perform challenge experiments in appropriate transgenic models.

Together the findings presented herein demonstrate nanoparticle carriers such as PSNPs which do not induce conventional inflammation, are capable of generating and enhancing CD8^+^ T cell immune responses, not just to model antigens in mice, but to vaccine relevant HLA-A2.1 restricted peptide epitopes from multiple proteins relevant to gynecological cancers. Furthermore, for specific peptide epitopes, PSNPs nanovaccines were shown to elicit CD8^+^ T cell responses even when other strong adjuvants failed to induce such responses. This study, however, suggests that for some particularly weak natural epitopes, neither conventional inflammatory adjuvants (CpG), or nanoparticle vaccine approaches may by themselves convert them into strong immunogens, and it will be necessary to optimize the use of super-agonist epitopes.

## Author Contributions

SX and MP: designed and supervised all experiments; SX: performed some of the experiments, analyzed and interpreted all the data; MP, AG, and AH: also analyzed and interpreted some of the data; KW: performed some of the experiments and analyzed some of the data; SX and MP: wrote the manuscript. All authors reviewed and agreed on the contents of the final version of the manuscript.

### Conflict of Interest Statement

SX and MP were the co-founding directors of the PX Biosolutions Pty Ltd who sponsored the research program presented here. The remaining authors declare that the research was conducted in the absence of any commercial or financial relationships that could be construed as a potential conflict of interest.
